# Epidemiology of atherosclerotic cardiovascular disease in polygenic hypercholesterolemia with or without high lipoprotein(a) levels

**DOI:** 10.3389/fcvm.2023.1272288

**Published:** 2024-01-22

**Authors:** Pierandrea Vinci, Nicola Fiotti, Emiliano Panizon, Letizia Maria Tosoni, Carla Cerrato, Federica Pellicori, Alessia Pirulli, Nicola Altamura, Paolo Schincariol, Filippo Giorgio Di Girolamo, Gianni Biolo

**Affiliations:** ^1^ U.C.O. Clinica Medica, Department of Medical, Surgical and Health Sciences, University of Trieste and ASUGI, Trieste, Italy; ^2^ Hospital Pharmacy, Cattinara Hospital, Azienda Sanitaria Universitaria Giuliano Isontina, Trieste, Italy

**Keywords:** lipoprotein(a), atherosclerotic cardiovascular disease, major adverse cardiovascular events, stroke, peripheral arterial disease, stable angina, hypercholesterolemia, atherosclerosis

## Abstract

**Background and aims:**

Epidemiology of atherosclerotic cardiovascular disease might be different in patients with polygenic hypercholesterolemia plus high levels (≥30 mg/dl) of Lp(a) (H-Lpa) than in those with polygenic hypercholesterolemia alone (H-LDL). We compared the incidence of peripheral artery disease (PAD), coronary artery disease (CAD), and cerebrovascular disease (CVD) in patients with H-Lpa and in those with H-LDL.

**Methods:**

Retrospective analysis of demographics, risk factors, vascular events, therapy, and lipid profile in outpatient clinical data. Inclusion criteria was adult age, diagnosis of polygenic hypercholesterolemia, and both indication and availability for Lp(a) measurement.

**Results:**

Medical records of 258 patients with H-Lpa and 290 H-LDL were reviewed for occurrence of vascular events. The median duration of follow-up was 10 years (IQR 3–16). In spite of a similar reduction of LDL cholesterol, vascular events occurred more frequently, and approximately 7 years earlier (*P* = 0.024) in patients with H-Lpa than in H-LDL (HR 1.96 1.21–3.17, *P* = 0.006). The difference was around 10 years for acute events (TIA, Stroke, acute coronary events) and one year for chronic ones (*P* = 0.023 and 0.525, respectively). Occurrence of acute CAD was higher in H-Lpa men (HR 3.1, 95% CI 1.2–7.9, *P* = 0.007) while, among women, PAD was observed exclusively in H-Lpa subjects with smoking habits (*P* = 0.009).

**Conclusions:**

Patients with high Lp(a) levels suffer from a larger and earlier burden of the disease compared to those with polygenic hypercholesterolemia alone. These patients are at higher risk of CAD if they are men, and of PAD if they are women.

## 1 Introduction

Atherosclerotic cardiovascular disease (ASCVD) is still a leading cause of morbidity and mortality, despite prevention and treatment strategies based on lifestyle modifications, and management of its risk factors, namely smoking habits, hypercholesterolemia, hypertension, diabetes, and obesity. Hypercholesterolemia is a heterogeneous condition encompassing two main disorders, namely familial hypercholesterolemia (FH) carrying LDL, LDLR, APOB, PCSK9- and LDLRAP1-related genetic defects, and polygenic hypercholesterolemia (PH) due to multiple genetic variants. Given the many genetic variants involved in its diagnosis, PH is diagnosed via primary hypercholesterolemia and negative genetic testing. While PH has a high prevalence in the population (1 out of 20 subjects) and accounts for the vast majority of atherosclerotic events, FH has a lower prevalence (1 out of 200–300 subjects) and a more severe and earlier occurrence (1–3).

Despite achieving the therapeutic goals for all cardiovascular risk factors in these patients, a residual risk persists; Lp(a) represents an emerging risk factor to explain such a discrepancy. Lp(a) is a circulating low-density lipoprotein (LDL) in which a large glycoprotein, apolipoprotein(a) [Apo(a)], is bound via a disulfide bridge to apo B100 (4). Circulating Lp(a) levels are genetically determined by the *LPA* gene encoding Apo(a), and remain stable during a lifetime (5), with limited response to lipid-lowering drugs. Epidemiological, genetic, and Mendelian randomization studies provide strong evidence that Lp(a) is also associated with the development of ASCVD, i.e., coronary artery disease (CAD), cerebrovascular disease (CVD), and peripheral artery disease (PAD) (6–10), beyond calcific aortic valve disease (CAVD) (11–13). Ex vivo or population studies show that high Lp(a) levels (or *LPA* polymorphisms) are associated with the development of plaques presenting features of vulnerability, namely lipid-rich necrotic core, thin or ruptured fibrous cap (14), and smooth muscle cell content (15). Also, in the same study, intraplaque hemorrhage and a higher degree of stenosis were observed more often in high Lp(a) women and men (16), respectively, when compared to plaques obtained from controls.

Association between high Lp(a) and FH is much higher than predicted by the prevalence of the genetic variants involved: while the prevalence of Lp(a) > 50 mg/dl is around 20% in the general European population, this figure rises to 29.2 (17) or 35.8% in FH (18). Such an association is less investigated in patients with PH, in spite of the higher contribution of these two conditions in the occurrence of ASCVD. Whether and how high Lp(a) levels modify the natural development and progression of atherosclerosis is not well defined. It can be hypothesized, therefore, that higher LP(a) levels are associated with more unstable or earlier patterns of atherosclerotic events. This study investigates the pattern of occurrence of vascular events according to Lp(a) levels (≥30 mg/dl) in a group of patients with polygenic hypercholesterolemia attending a secondary level outpatient clinic in Northern Italy. In particular, we investigated whether there was a correlation between timing and frequency of occurrence of vascular event and Lp(a) levels, and whether the interaction between Lp(a) and gender could promote different settings of vascular events in specific vascular districts.

## 2 Patients and methods

### 2.1 Study population

A single-center review of medical records was carried out in patients with PH attending a secondary level outpatient clinic (Clinica Medica Generale, ASUGI) from a University Hospital in the North-East of Italy. The center follows 3,600 patients yearly with different dyslipidemic disorders. All records of patients with indication to Lp(a) measurement according to the criteria identified by the consensus panel of the European Atherosclerosis Society (19) were selected. Namely, these criteria were: ≥5% 10-year risk of fatal ASCVD according to the European guidelines (20), patients with premature ASCVD, a family history of premature ASCVD with or without elevated Lp(a), and recurrent ASCVD despite statin treatment. Only patients with an in-house measurement of Lp(a) were considered. Venous blood samples were obtained at the time of admission to the Clinics according to the previously published protocol (21) and analyzed according to the standard methods in the central laboratory of the Hospital of Cattinara (Trieste, ITALY). The dosage of Lp(a), total cholesterol, LDL-cholesterol, HDL-cholesterol, and triglycerides is expressed in mg/dl. The Lp(a) dosage was obtained by (isoform independent) immunoturbidimetric method with U-5800 Beckman analyzer.

The observation period for each patient started either with the index event or with the enrollment at the Center and ended on October 1st 2022.

Clinical data concerning arterial vascular events (type and timing), and cardiovascular risk factors as hypertension, diabetes mellitus (DM), smoking, and family history of early (<60 if women, or <55 if man) coronary artery event in a first degree relative were considered. The definition of these conditions followed those previously published (21). The diagnosis of PH was set according to the recommendations in force at the time of determination (22). All subjects were screened with the Dutch Lipid Clinic Network criteria, which assigns points to risk factors in order to stratify the likelihood of diagnosis of familial hypercholesterolemia into one of four categories: definite (>8), probable (6–8), possible (3–5), and unlikely (<3), so FH was defined as a score ≥6 plus the positive result of a genetic test for LDLR, APOB, PCSK9, and LDLRAP1 (23, 24), while PH was diagnosed in the remaining patients with negative genetic testing results, independent of the Dutch Lipid Score. Furthermore, exclusion criteria were an age of <18 years old, genetic diagnosis of heterozygous or homozygous FH, and patients with secondary hyperlipidemia or an incomplete medical record. Written informed consent was obtained from all patients; the study complies with the 1975 Helsinki declaration and approval of the ethical committee was previously obtained (21).

**Figure 1:**
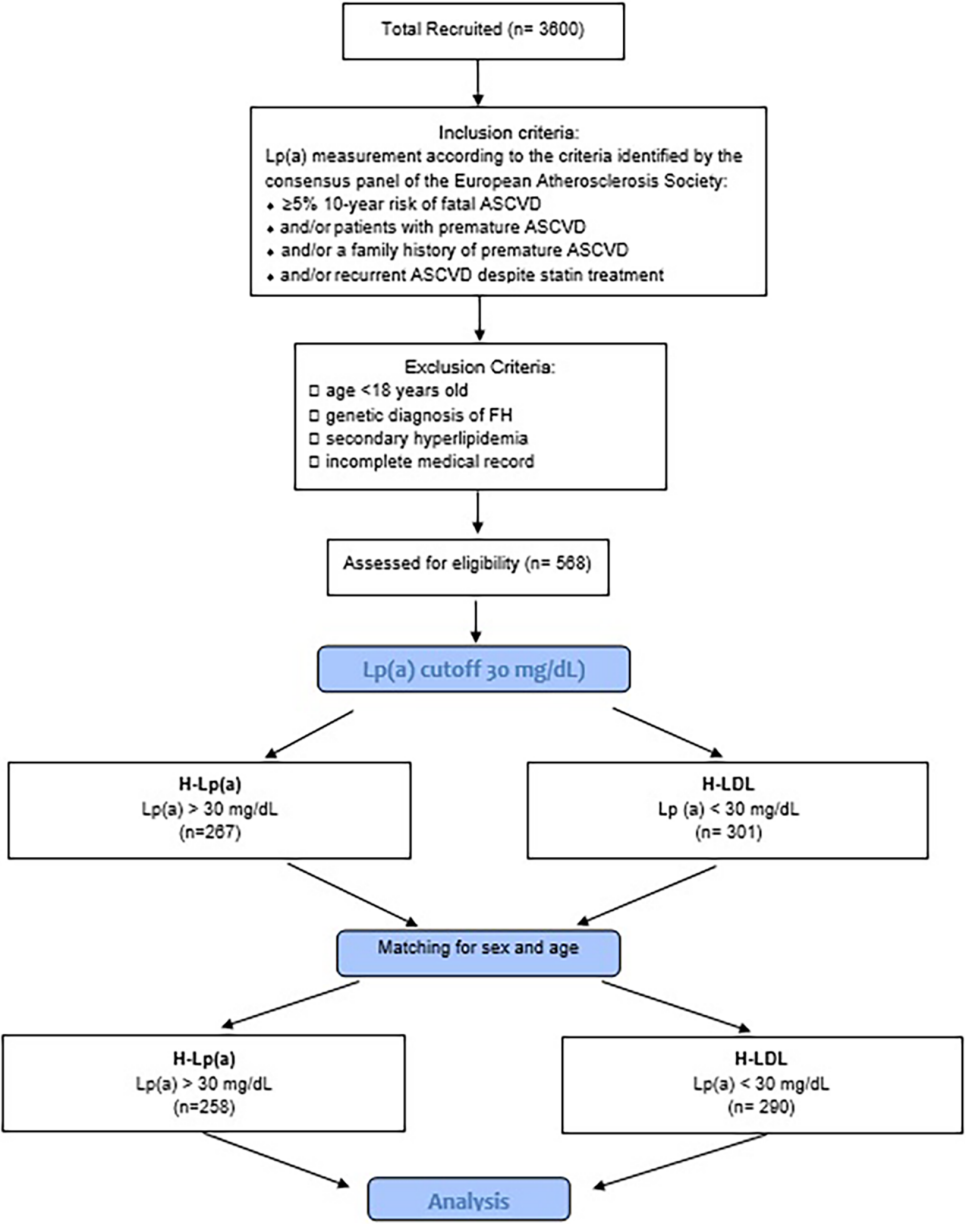
Flow-chart of the study. H-Lpa: patients with hypercholesterolemia plus Lp(a) levels >30 mg/dl; H-LDL: patients with polygenic hypercholesterolemia alone.

During the attendance at the center, the clinic enforced lifestyle changes and optimized lipid lowering drugs with regular follow-up examinations and monitoring of lipid levels. Any patient at high or moderately high risk who had lifestyle-related risk factors (e.g., obesity, physical inactivity, elevated triglyceride, low HDL-C, or metabolic syndrome) was considered as a candidate for therapeutic lifestyle changes regardless of LDL-C level. Only those patients who did not reach the therapeutic target after this primary approach were treated with lipid lowering drugs. For investigation of the effects of Lp(a), the patients with PH were split into 2 groups: one of 258 patients with hypercholesterolemia plus Lp(a) levels ≥ 30 mg/dl (H-Lpa) and another of 290 patients with polygenic hypercholesterolemia alone (H-LDL) after matching the two groups for age and sex (Figure 1).

This study has been conducted in accordance with the World Medical Association's Declaration of Helsinki and an informed written consent was obtained from all participants.

This study involving human participants was reviewed and approved by CEUR (Comitato Etico Unico Regionale Fvg) number 2017-Os-040-ASUITS.

### 2.2 Methodology of assessment of vascular events (ASCVD)

All those events occurring until the age of 75 for each patient and reported in a standardized Case Report Form were considered. Events had to be either documented (hospitalization or clinical records) or self-reported but, in the latter case, confirmed by a trained health care professional at the time of the report.

Trigger events leading to enrollment in the Center were also retrieved. In detail, the events considered were those encompassed by ASCVD (25), i.e., CAD (ischemic cardiopathy, myocardial infarction and its acute complications, coronary artery bypass graft surgery, or percutaneous angioplasty/stent placement), ischemic stroke (cerebral infarction due to thrombosis or cerebral atherosclerosis or cerebrovascular syndromes), transient ischemic attack (TIA), carotid thromboendarterectomy (TEA), and PAD. To increase detail on the pathophysiology of Lp(a), analysis was further divided into acute (myocardial infarction and its acute complications, cerebral infarction, TIA) and chronic vascular events (ischemic heart disease, coronary artery bypass graft surgery or percutaneous angioplasty/stent placement, carotid thromboendarterectomy, and PAD).

### 2.3 Statistical analysis

Data was anonymized before handling. Continuous variables are described as median and interquartile values (IQR), while categorical variables are described as the absolute count and percent value within the group. The comparison of continuous variables between the two groups was conducted according to non-parametric statistics, i.e., with Mann–Whitney and Kruskal–Wallis tests for the comparison of continuous variables between different categories. The association between two dichotomous or categorical variables was tested with a c^2^ test and Hazard Ratio with 95% confidence interval (95% C.I.). Changes of lipid parameters between first and last observation were compared with a generalized linear model test for repeated measurements, while also considering the interaction with the Lp(a) levels. In addition, Kaplan-Meier curves were used to determine the time course of events during follow-up. To assess the importance of sex in the occurrence of events, Cox proportional hazards statistics were carried out for PAD or acute coronary events. Independent variables included in this analysis were high Lp(a) (1 = yes 0 = no), Dutch score (0–2 unlikely, 3–5 possible, 6–8 likely, >8 definite), smoking habit (0 = never, 1 = ever a smoker), Hypertension, Diabetes, and family history of early CV disease. To observe the effect of increasing Lp(a) levels on the incidence of events, the Lp(a) group was divided according to classes (0 = 30–50 1 = 50–100 2 = 100–150 3 = >150 mg/dl) and in patients below and above the median value (87 mg/dl). All analyses were conducted using Statistical Package for Social Sciences (SPSS) 21.0 software (SPSS Chicago, IL). Statistical significance was considered for *P* values of <0.05.

## 3 Results

For this study, the medical records of 548 patients were available, 258 with high Lp(a) levels (>30 mg/dl) (H-Lpa) and 290 dyslipidemic patients (H-LDL) with normal circulating levels of Lp(a) (<30 mg/dl). The median duration of follow-up was 10 years (IQR 3–16).

In H-Lpa patients, the prevalence of diabetes and body mass index (BMI) was significantly lower compared to the H-LDL group. To rule out the interferences of diabetes on BMI, sub-analysis was conducted in non-diabetic patients: lower BMI was observed only in non-diabetic H-Lpa women (*P* = 0.037). Among the remaining common atherosclerosis risk factors, hypertension (33%) and smoking habits, either current or past, had similar prevalence (34 vs. 39%). H-Lpa patients reported a significantly higher prevalence of premature cardiovascular events in the family history (*P* = 0.038). Other lipid biomarkers (total cholesterol, LDL cholesterol, triglycerides) were significantly higher among H-LDL subjects. Clinical and biochemical characteristics of the two groups (H-Lpa and H-LDL patients) are summarized in Table 1.

**Table 1:** Clinical, epidemiological, and laboratory characteristics of the study group, divided according to the type of dyslipidemia.

	H-Lpa	H-LDL	*P*-value
Baseline			
Number	258	290	
Sex, M/F	101 (39%)/157 (61%)	127 (44%)/163 (56%)	0.271
Age at diagnosis	49 (39–58)	48 (37–57)	0.745
Months of follow up	121 (45–196)	140 (58–240)	0.161
BMI, kg/m^2^	24.7 (22.6–28)	25.7 (23–28,3)	0.032
Risk factors			
Hypertension	85 (33%)	96 (33%)	0.969
Diabetes mellitus	6 (2%)	21 (7%)	0.008
Smoking habit			0.331
Current smoker	30 (12%)	45 (16%)	
Former smoker	57 (22%)	68 (23%)	
Non smoker	171 (66%)	177 (61%)	
Positive family history	72 (28%)	59 (20%)	0.038
Lipid profile			
Total cholesterol (mg/dl)*	234 (196–267)	255 (211–294)	<0.001
HDL-cholesterol (mg/dl)	59 (49–70)	55 (47–69)	0.071
LDL-cholesterol (mg/dl)**	148 (120–183)	165 (129–194)	0.005
Triglycerides (mg/dl)***	104 (79–146)	133 (96–200)	<0.001
Lipoprotein(a) (mg/dl)	86.6 (60–122)	8 (4–15)	–
DUTCH lipid score			*0.584*
Unlikely (0–2)	141 (55%)	145 (50%)	
Possible (3–5)	84 (33%)	110 (38%)	
Probable (6–8)	18 (7%)	21 (7%)	
Definite (>8)	15 (6%)	14 (5%)	
Therapy (number of subjects)			
Statins	103	78	
Integrators	25	27	
Ezetimibe	37	26	
n-3	8	9	
Fibrates	3	0	
End of follow-up			
Lipid profile			
Total cholesterol (mg/dl)*	184 (159–211)	192 (161–220)	.102
HDL-cholesterol (mg/dl)	59 (49–69)	55 (46–65)	.026
LDL-cholesterol (mg/dl)**	103 (80–125)	106 (87–135	.057
Triglycerides (mg/dl)***	94 (73–128)	115 (81–159)	<0.001
Therapy (number of subjects)			
Statins	143	188	
Integrators	26	0	
Ezetimibe	88	102	
n-3	13	70	
Fibrates	3	4	
PCSK9 inhibitors	7	2	

Data are reported as absolute values and percentage within the group or median and interquartile values, all values of lipid profile are expressed in mg/dl. Positive family history = positive history cardiovascular event in a first degree relative at young age (<60 for women, or <55 for men). DUTCH lipid score = assigns points to risk factors and stratifies likelihood of diagnosis of familial hypercholesterolemia into one of four categories: definite (>8), probable (6–8), possible (3–5), and unlikely (<3). Therapy = number of patients assuming the specific lipid-lowering drug at baseline (at arrival at the lipid clinics) and at the end of follow-up.

* Generalized linear model *P* < .001 for the whole population and.054 between groups.

** *P* < .001 for the whole population and .654 between groups.

*** *P* < .001 for the whole population and .006 between groups.

Concerning lipid-lowering drugs, statins were the most widely prescribed at the end of the follow-up: 57% H-Lpa and 65% H-LDL subjects were on statin therapy and the latter group used higher intensity principles or dosages of these drugs (*P* < 0.001). At enrollment, patients not assuming statins had significantly higher Lp(a) levels compared to those who did (80,5 IQR 53,3–118 vs. 13, IQR 5–52, *P* < .001). Such a difference did not correlate with higher prevalence of events (*P* = 0.424). Other low-density lipoprotein (LDL)-lowering drugs that were used included ezetimibe, with higher prevalence in H-Lpa patients at the end of the follow-up (46.3 vs. 35.3%, *P* = 0.016), PCSK9 inhibitors were more frequently used in H-Lpa (3.7 vs. 0.7, *P* = 0.019), and lipoprotein apheresis were used in two high-risk patients for secondary prevention (both cases). Considering the effect of PCSK9 on Lp(a), a sub-analysis has been conducted on patients not assuming these drugs. The association between high Lp(a) levels and incidence of events has been substantially confirmed (*P* = 0.016). All patients, however, received a pharmacologic treatment for their condition. When the last lipid test for each patient was compared to baseline, there was a significant reduction in total LDL cholesterol and triglycerides in the whole population, with a difference between the two groups observed only for Triglycerides (*P* < .001). HDL cholesterol did not change significantly over time.

**Figure 2:**
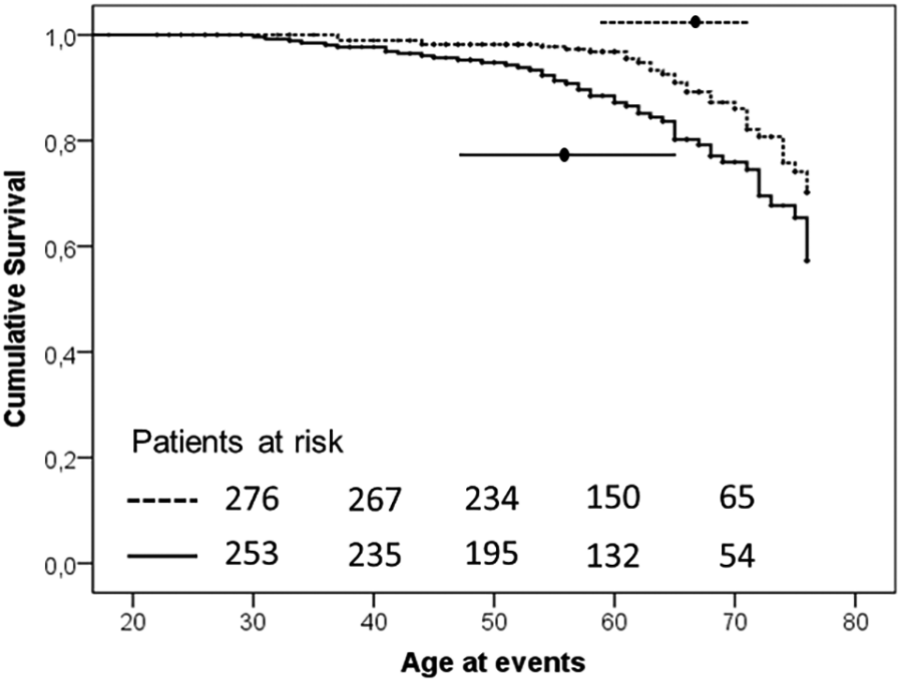
Kaplan–Meier curve of occurrence of ischemic ASCVD events in the two study groups (*P*-value = .003). Horizontal lines represent median and interquartile values for age at the time of the events (*P* = .024, Mann–Whitney test). Solid line = H-Lpa and dotted line = H-LDL.

Vascular events have been observed approximately 7 years earlier in H-Lpa patients compared with H-LDL (57.5 IQR 47–65 vs. 65, IQR 58–71, *P* = 0.024). The associations of Lp(a) on the age and the burden of events were also confirmed with a Kaplan–Meier survival curve (Figure 2). The cumulative prevalence of all events was 1.49 100 pt/y in H-Lpa compared to 0.75 100 pt/y in H-LDL. When a higher cut-off Lp(a) level of 50 mg/dl was adopted, the results were confirmed but with a lower sensitivity (*P* = 0.007), (data not reported).

**Figure 3:**
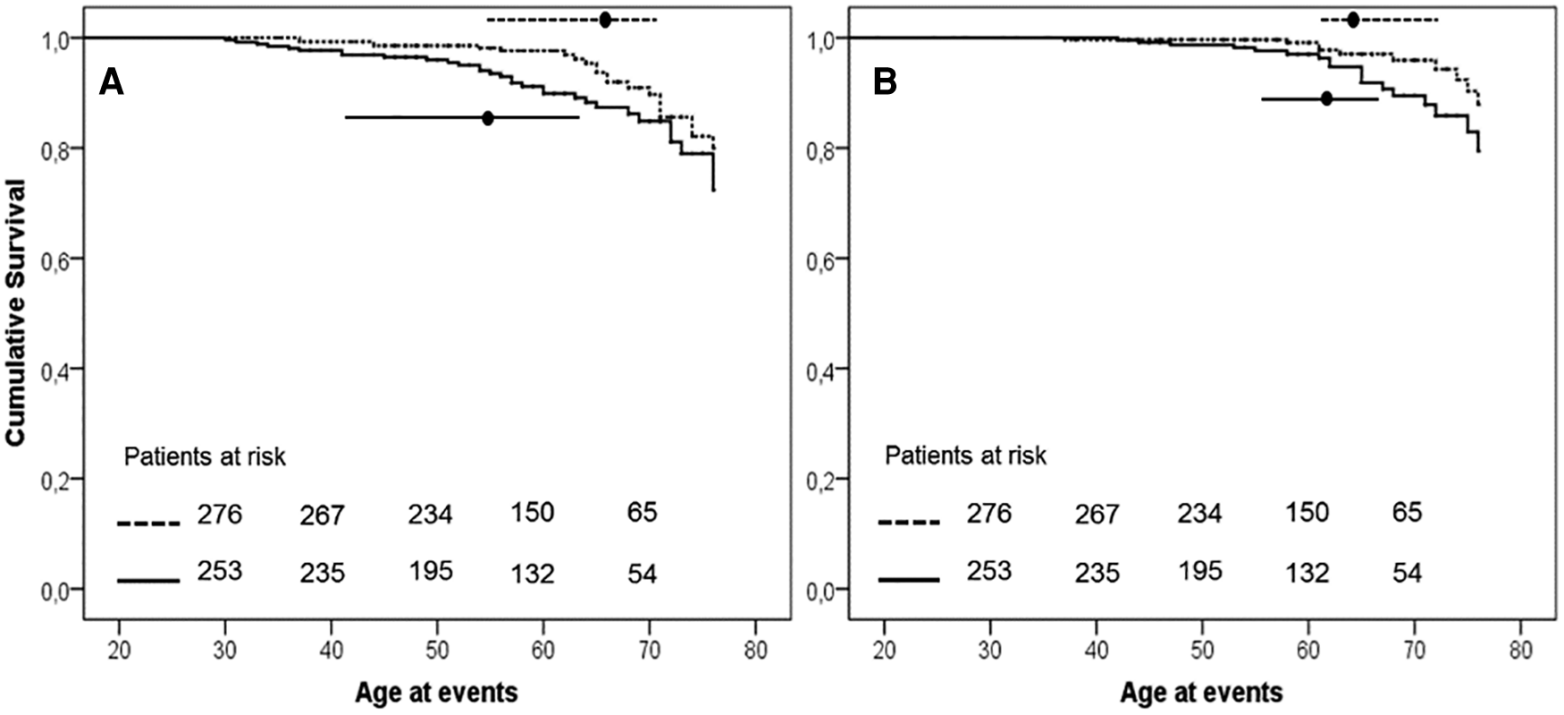
Event free survival curve Kaplan–Meier curve for occurrence of acute (**A**, unstable angina, acute myocardial infarction, TIA, or stroke) or chronic (**B**, stable angina, transcutaneal endatherectomy TEA, PAD) ischemic ASCVD events. *P* value of Kaplan–Meier curve.036 and .037, for panels **A** and **B**, respectively. Horizontal lines represent median and interquartile values for age at the time of the events (*P* = .023 and .525 Mann–Whitney test for comparisons of acute and chronic events, respectively). Solid line = H-Lpa, dotted line = H-LDL.

The earlier occurrence of events in H-Lpa was confirmed for the acute cases (i.e., TIA, Stroke, acute coronary events,) with patients who were 55 years old, IQR 41–63, vs. 65 years old, IQR 55–71, in H-Lpa and H-LDL, respectively (*P* = 0.023). On the contrary, chronic events (stable angina, TEA, or PAD) occurred at approximately 62 years old (IQR 55–67), and 63 years old (IQR 61–72) respectively (*P* = 0.525). Such a difference was confirmed with a Kaplan–Meier curve as in Figure 3. Within the group of H-Lpa patients, the incidence of events did not differ according to the Class of Lp(a) or according to median value (data not reported).

**Figure 4:**
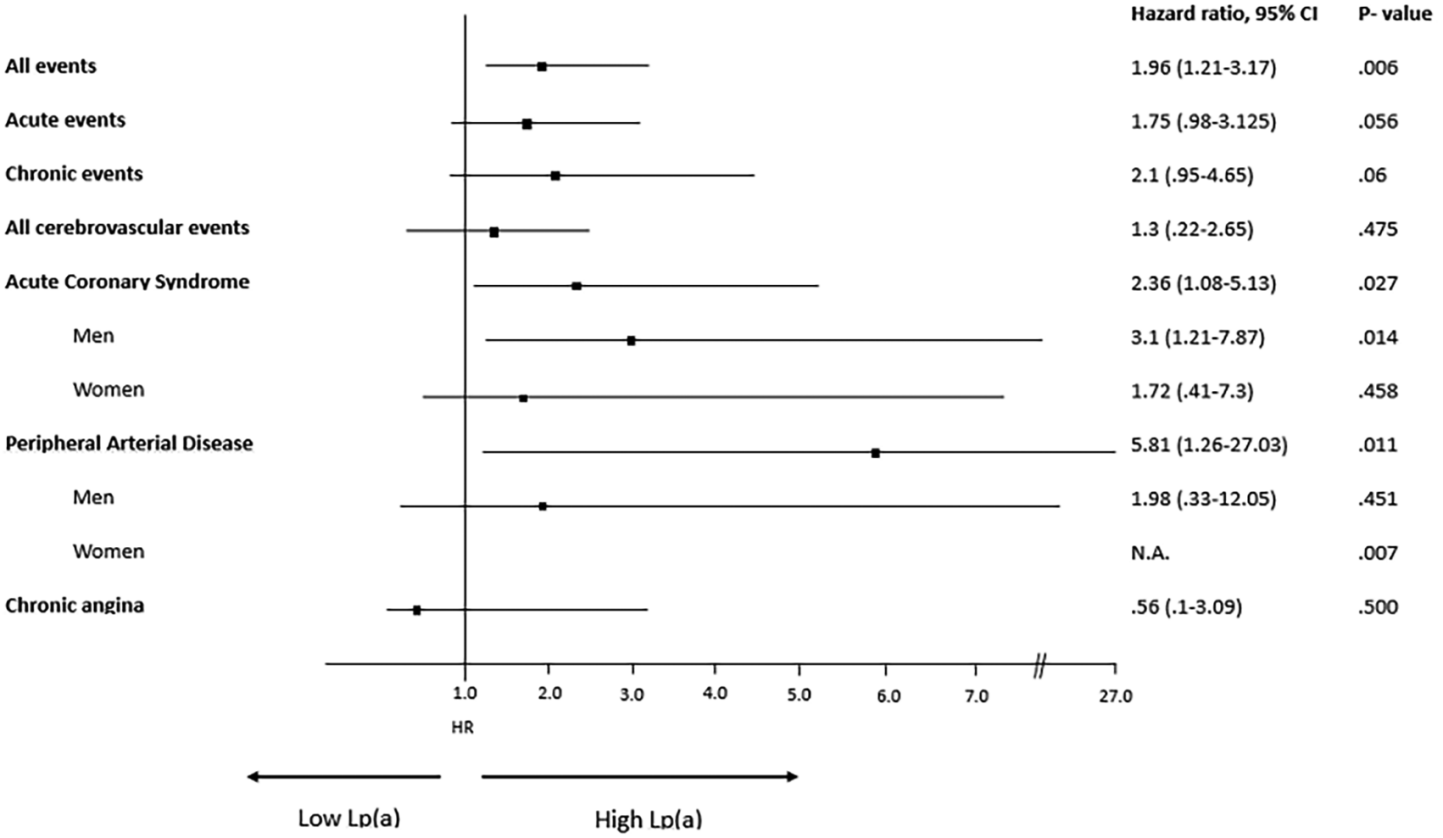
Forrest plot, hazard ratio, 95% confidence intervals (C.I. 95%) and *χ*^2^ *P* value of different vascular events according to high (≥30 mg/dl) or low (<30 mg/dl) Lp(a) levels. When relevant, the analysis is split according to sex. N.A.= not applicable due to 0 events in H-LDL women. HR for men and women have been reported separately only when different between the two sexes.

**Figure 5:**
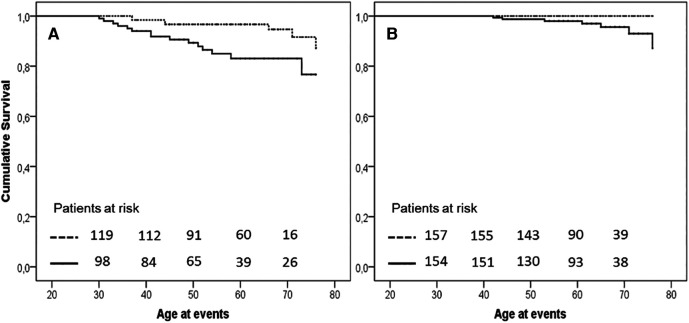
Kaplan–Meier curve of occurrence of acute coronary events in men (panel **A**, *P* = .007) and of PAD in women (panel **B**, *P* = .009) in the two study groups. Solid H-Lpa and dotted H-LDL.

A description of each type of recorded vascular events, in total and divided by sex, is detailed in Table 2 and Hazard Ratio is described in Figure 4. Among the different vascular diseases, coronary events and PAD account for differences between H-Lpa and H-LDL, in particular ACS in men and PAD in women (Figure 5). In this latter case, Hazard Ratio and 95% C.I. could not be calculated due to 0 (zero) events in H-LDL women.

**Table 2:** Prevalence of vascular events in the two study groups.

Events	H-LPA			H-LDL					
	All	Men	Women	All	Men	Women	P	*P* men	*P* women
Number	258			290					
All	49	26	23	31	21	10	.006	.021	.019
Acute	31	19	12	21	13	8	.056	.026	.301
Chronic	18	7	11	10	8	2	.06	.405	.031
All cerebrovascular	17	6	11	15	9	6	.475	.78	.2
ACS	20	15	5	10	7	3	.027	.014	.458
PAD	10	3	7	2	2	0	.011	.451	.007
Chronic angina	2	1	1	4	3	1	.500	.572	.992

ACS, acute coronary syndrome; PAD, peripheral arterial disease; *P*-value = *χ*^2^ square test; *P* men = *P* value of *χ*^2^ test among men; *P* women = *P* value of *χ*^2^ test among women.

### 3.1 Cox regression analysis

Cox regression analysis investigated the interaction and association of Lp(a) with other known risk factors in the occurrence of PAD and of acute coronary syndromes. Acute coronary syndrome was more frequent in men with high Lp(a) and with a higher Dutch score, while in the analysis of PAD, the role of Lp-a could be demonstrated in women separated from men. The results are summarized in Table 3.

**Table 3:** Final steps of Cox proportional hazards analysis of risk factor for the age of diagnosis of ACS and PAD according to sex and Lp(a).

	B	SE	Wald	Sig.	Exp(B)
Acute coronary syndrome in men and women					
SEX (male = 1, female = 0)	1.953	.467	17.51	.000	.142
LDL cholesterol	-.017	.006	8.928	.003	.983
Lp(a) (mg/dl)	.007	.003	4.663	.031	1.007
Dutch score	.393	.184	4.568	.033	1.482
Peripheral arterial disease in women					
Lp(a) cutoff ≥30 mg/dl (<30 mg/dl = 1, ≥30 mg/dl = 2)	4.712	2.714	3.015	.083	.009

Independent variables included in this analysis were high LDL (1 = yes 0 = no), high Lp(a) (1 = yes 0 = no), Dutch score (0–2 unlikely, 3–5 possible, 6–8 likely, >8 definite), smoking habit (0 = never, 1 = ever a smoker), Hypertension (1 = yes 0 = no), Diabetes (1 = yes 0 = no), and family history of early Cardiovascular disease (1 = yes 0 = no).

## 4 Discussion

Lp(a) can start and complicate atherosclerosis with different putative mechanisms (26, 27) and Hyperlipoproteinemia (a) represents an independent risk index for ASCVD (26). Investigation on the relationship between Hyperlipoproteinemia (a) and Hypercholesterolemia has the potential to improve the management of cardiovascular risk. The main results of the present study are that, (a) the degree of association of high Lp(a) (>30 mg/dl) with polygenic hypercholesterolemia is higher than suspected; (b) that high Lp(a) worsens the pattern of vascular events, and that (c) such a risk is different according to sex and vascular district.

The first result is that Polygenic hypercholesterolemia is closely associated with increased Lp(a) levels in this study: while the prevalence in the general population is reported around 20%, in our study group it is well above 50%. Even considering a lower cutoff of 30 mg/dl, this cannot be attributed to chance. Such an association was already observed in familial hypercholesterolemia and in other combined forms of hypercholesterolemia (28). This finding can be attributed to a genuine association or to an increased likelihood of observing polygenic hypercholesterolemia in patients with high Lp(a) as in the conclusions of Trinder (18).

Our study also demonstrates that the risk of vascular events in high Lp(a) patients doubled, and events occur approximately 7 years earlier when compared with those observed in polygenic hypercholesterolemia. The quality of our follow-up for high Lp(a) patients is close to that observed in other specialized centers, even using Lp(a) apheresis (29) (1.49 vs. 1.5 100pt/year). The group of acute events, assumed to derive from rupture or erosion of the plaques, occurred (marginally) more often and earlier in Lp(a) patients than in PH alone, while the age of onset and prevalence were similar in those events related to progressive chronic growth of plaques (PAD, stable angina, and TEA). The increased prevalence of events in Hyper Lp(a) has also been observed by Schatz et al, on patients with a history of premature cardiovascular events (30). Our results corroborate Schatz's findings, expanding the comparison of Hyper Lp(a) to patients with polygenic hypercholesterolemia and investigating the onset of all acute and chronic coronary and cerebral events, as well as peripheral arterial disease. Furthermore, consistent with the cited work, a 40% increase in a positive family history of vascular events was found in the present study in patients with elevated Lp(a). Other studies have observed earlier acute events in high Lp(a) within a court of early coronary heart disease (31), but not in comparison with FH (32). Our experience agrees with these two observations, since our comparator, polygenic hypercholesterolemia, is less severe and less precocious than FH.

The starting hypothesis, i.e., that acute events should prevail in carriers of high levels of Lp(a), is confirmed in the present study. This finding has a parallel in some histopathology studies showing more prevalent features of instability, namely thin-cap fibroatheroma lipid-rich necrotic core and stenosis, in patients with high Lp(a) (14–16). The evidence that chronic events such as PAD also occur earlier in hyper Lp(a) may be explained by the finding of unstable plaques (calcified, with intraplaque hemorrhage and disrupted) in 72% of common iliac arteries of patients without symptoms of PAD (33). This suggests that the progression of PAD may also occur through accumulation of small acute events in large arteries.

The third main result is that the interaction between Lp(a) and gender promotes a different setting of vascular events. In the present study we observe a significant relationship between high lipoprotein (a) and acute coronary events, mainly in men. This evidence is confirmed by the Cox analysis, which identifies male sex, LDL cholesterol, Lp(a) and Dutch Lipid score as variables which can predict coronary events. The higher prevalence of events in male patients could be explained by assuming that some features of plaque instability are more frequent in men than in women. In fact, the study of Van Dam-Nolen and coworkers reports that stenotic plaques are more prevalent in men with high Lp(a) than in women (34). It could be hypothesized that constrictive remodeling of plaques in men triggers events in coronary arteries, since they are the smallest vessels investigated.

Furthermore, we have identified a connection between PAD and women with high Lp(a). In literature, smoking and type 2 diabetes mellitus are classically associated with the development of PAD (35, 36), although observational studies show that higher Lp(a) concentrations are also an independent risk factor for this condition (37). Such an association has been found in some studies (38–40) while others did not achieve such a result (41, 42). In our study, among women, PAD is observed exclusively in women who were (current or former) smokers and with high Lp(a). The Cox regression analysis only partially confirmed the univariate results, probably because of a lower event rate for this complication. The most likely explanation of this evidence showing a role of Lp(a) only in women is that some plaque features in patients with high Lp(a) are preferentially observed in this sex and explain the progression of PAD. This situation has been described in the study of van dam Nolen, which reports that intraplaque hemorrhages have been observed more frequently in female patients with high Lp(a) (16). How Lp(a) can support the development and progression of PAD through intraplaque hemorrhage can only be conjectured. Ex vivo studies show that Lp(a) enhances angiogenesis in the chorioallantoic membrane (43) and this could synergize with the development of an abnormal angiogenesis observed in smoking (44) leading to an enhanced plaque development. This pathophysiology might lead to increased bleeding into plaques and contribute to the progress of atherosclerosis in large arteries such as those supplying the lower limbs. Another important point, overturning all naïve concepts, is that the prevalence of vulnerability characteristics changes over time and some associations might be lost across long term observations (45). These changes are particularly important in women, whose hormonal pattern evolution affects the immune, inflammatory, and coagulative response.

Finally, differences in prevalence of lipid-lowering drug use and diabetes observed in the two populations need to be highlighted and commented on. The lower intensity of statins in Lp(a) patients in our study (with minimal modifications during follow-up) is in accordance with the recommendations of Tsimikas et al, reporting a possible increase in Lp(a) levels during higher intensity statins (46) and therefore suggesting a reduction in their intensity. Indeed, there is no general agreement on this effect, with some studies showing a 10 to 20% increase in Lp(a) (46), and others not observing this result (47, 48). This difference in intensity was associated with a similar pattern of lipid profile modifications, but the incidence of events remained higher in Lp(a). Whether this is due to an intensified influx and adhesiveness of Lp(a)-LDL particles into the vessel matrix (subendothelium) or insufficient pleiotropic of statins is not known and deserves further investigation, such as adding another arm in the comparison, namely AntiSense Oligonucleotide and siRNA targeting *LPA*. One speculation on the early age of events in a comparable dyslipidemia (LDL-cholesterol) setting is that Lp(a) might hinder an antiatherogenic mechanism which could be the release of LDL complex from the subendothelial layer of lesion prone arteries. This implies a dynamic trafficking of LDL within the lesions, as demonstrated by Jagannathan et al. (49), and Lp(a) acting as an element favoring the permanence of LDL within the growing plaque.

Another point is that Lp(a) lowers the risk of diabetes (50, 51), and therefore fewer patients with high Lp(a) and diabetes were expected (and actually found) in our study. This difference definitely hinders a precise comparison of the two populations and might be recognized as a confounder, although this result reflects the accuracy of patient selection. Since diabetes is a major contributor to vascular atherosclerotic burden, its reduced prevalence in high Lp(a) patients should have also reduced the prevalence of events, while the opposite has been observed. M. Kostner et al. calls this phenomenon “The Lp(a) Paradox in Diabetes Mellitus” (52): patients with Type-2 diabetes mellitus (T2DM) may have reduced Lp(a) due to mutations or polymorphisms in genes that affect the expression of the APOA gene, and it could explain the evidence that T2DM patients have lower Lp(a) plasma levels in comparison to individuals without T2DM.

The choice of a cutoff level at 30 mg/dl requires some explanation. In spite of the ESC/EAS recommendation of a cutoff value of 50 mg/dl, the literature has not unanimously identified such an Lp(a) value as critical for the increase in cardiovascular risk (53–57). Indeed, we also assessed the cutoff value of 50 mg/dl (data not reported) but the results were consistent with those obtained with the lower cut off at 30 mg/dl and, moreover, included 31 more subjects and 5 events (3CVD, 1 TIA and 1 Stroke) in the HLp(a) patients, which increased the sensitivity of the comparison.

Some limitations of the present study have to be acknowledged. A large multicenter prospective study would have granted sub-analysis of other conditions, although the present report (encompassing polygenic hypercholesterolemia) is one of the largest in literature. Although a prospective study investigating the general population would have provided a more complete picture of the Lp(a) epidemiology, it would have required a follow-up of decades and significant financial and logistic support. Another potential limitation is that no fatal events were recorded. The effect of fatal events on the overall vascular burden is not well established for Lp(a), but with a fatality rate of cerebral and coronary vascular events under 10% in different studies, the inclusion of fatal events would have probably changed the final figures, but with only a small effect on the results of the present study. Furthermore, we did not reassess Lp(a) plasma levels following statins, mainly because guidelines in force during the follow-up did not recommend repeating such a measurement and the immunoturbidimetric method is not influenced by the different Lp(a) isoforms (58, 59).

In conclusion, this retrospective case-control study shows a significantly increased risk of ASCVD events in patients with high Lp(a) levels in comparison with patients with polygenic hypercholesterolemia. Vascular events also occur earlier in high Lp(a) patients, specifically, the incidence of acute CAD events was higher in high Lp(a) men, and that of PAD events was higher in high-Lpa smoker women.

## Data Availability

The raw data supporting the conclusions of this article will be made available by the authors, upon reasonable request.

## References

[1] Paththinige CS Sirisena ND Dissanayake V. Genetic determinants of inherited susceptibility to hypercholesterolemia—a comprehensive literature review. Lipids Health Dis. (2017) 16(1):103. 10.1186/s12944-017-0488-4 28577571 PMC5457620

[2] Wilson PWF Polonsky TS Miedema MD Khera A Kosinski AS Kuvin JT. Systematic review for the 2018 AHA/ACC/AACVPR/AAPA/ABC/ACPM/ADA/AGS/APhA/ASPC/NLA/PCNA guideline on the management of blood cholesterol: a report of the American College of Cardiology/American Heart Association Task Force on clinical practice guidelines. J Am Coll Cardiol. (2019) 73(24):3210–27. 10.1016/j.jacc.2018.11.004 30423394

[3] Mach F Baigent C Catapano AL Koskinas KC Casula M Badimon L 2019 ESC/EAS guidelines for the management of dyslipidaemias: lipid modification to reduce cardiovascular risk. Eur Heart J. (2020) 41(1):111–88. 10.1093/eurheartj/ehz455. Erratum in: *Eur Heart J*. (2020) 41(44):4255. PMID: 31504418. 31504418

[4] Frank S Durovic S Kostner GM. The assembly of lipoprotein lp(a). Eur J Clin Invest. (1996) 26(2):109–14. 10.1046/j.1365-2362.1996.112255.x 8904519

[5] Enkhmaa B Anuurad E Berglund L. Lipoprotein (a): impact by ethnicity and environmental and medical conditions. J Lipid Res. (2016) 57(7):1111–25. 10.1194/jlr.R051904 26637279 PMC4918859

[6] Bucci M Tana C Giamberardino MA Cipollone F. Lp(a) and cardiovascular risk: investigating the hidden side of the moon. Nutr Metab Cardiovasc Dis. (2016) 26(11):980–6. 10.1016/j.numecd.2016.07.004 27514608

[7] Cesari M Pahor M Bartali B Cherubini A Penninx BW Williams GR Antioxidants and physical performance in elderly persons: the invecchiare in chianti (InCHIANTI) study. Am J Clin Nutr. (2004) 79(2):289–94. 10.1093/ajcn/79.2.289 14749236

[8] Yanaka K Akahori H Imanaka T Miki K Yoshihara N Kimura T Relationship between lipoprotein(a) and angiographic severity of femoropopliteal lesions. J Atheroscler Thromb. (2021) 28(5):555–61. 10.5551/jat.56457 32863296 PMC8193776

[9] Sun D Zhou BY Zhao X Li S Zhu CG Guo YL Lipoprotein(a) level associates with coronary artery disease rather than carotid lesions in patients with familial hypercholesterolemia. J Clin Lab Anal. (2018) 32(7):e22442. 10.1002/jcla.22442 29603377 PMC6817237

[10] Kotani K Yamada S Yamada T Taniguchi N Sakurabayashi I. The relationship between oxidized lipoprotein(a) and carotid atherosclerosis in asymptomatic subjects: a comparison with native lipoprotein(a). Lipids Health Dis. (2011) 10:174. 10.1186/1476-511X-10-174 21970553 PMC3196711

[11] Kamstrup PR Tybjærg-Hansen A Nordestgaard BG. Extreme lipoprotein(a) levels and improved cardiovascular risk prediction. J Am Coll Cardiol. (2013) 61(11):1146–56. 10.1016/j.jacc.2012.12.023 23375930

[12] Clarke R Peden JF Hopewell JC Kyriakou T Goel A Heath SC Genetic variants associated with lp(a) lipoprotein level and coronary disease. N Engl J Med. (2009) 361(26):2518–28. 10.1056/NEJMoa0902604 20032323

[13] Nordestgaard BG Chapman MJ Ray K Borén J Andreotti F Watts GF Lipoprotein(a) as a cardiovascular risk factor: current status. Eur Heart J. (2010) 31(23):2844–53. 10.1093/eurheartj/ehq386 20965889 PMC3295201

[14] Kato A Kinoshita D Nagata T Asakura K Katamine M Katsura A Lipoprotein (a) levels and vulnerable characteristics in nonculprit plaque in patients with acute coronary syndrome. Int J Cardiol Heart Vasc. (2022) 43:101120. 10.1016/j.ijcha.2022.101120 36118156 PMC9474856

[15] Verwer MC Waissi F Mekke JM Dekker M Stroes ESG de Borst GJ High lipoprotein(a) is associated with major adverse limb events after femoral artery endarterectomy. Atherosclerosis. (2022) 349:196–203. 10.1016/j.atherosclerosis.2021.11.019 34857353

[16] van Dam-Nolen DHK van Dijk AC Crombag GAJC Lucci C Kooi ME Hendrikse J Lipoprotein(a) levels and atherosclerotic plaque characteristics in the carotid artery: the plaque at RISK (PARISK) study. Atherosclerosis. (2021) 329:22–9. 10.1016/j.atherosclerosis.2021.06.004 34216874

[17] Alonso R Andres E Mata N Fuentes-Jiménez F Badimón L López-Miranda J Lipoprotein(a) levels in familial hypercholesterolemia: an important predictor of cardiovascular disease independent of the type of LDL receptor mutation. J Am Coll Cardiol. (2014) 63(19):1982–9. 10.1016/j.jacc.2014.01.063 24632281

[18] Trinder M DeCastro ML Azizi H Cermakova L Jackson LM Frohlich J Ascertainment bias in the association between elevated lipoprotein(a) and familial hypercholesterolemia. J Am Coll Cardiol. (2020) 75(21):2682–93. 10.1016/j.jacc.2020.03.065 32466883

[19] Kronenberg F Mora S Stroes ESG Ference BA Arsenault BJ Berglund L Lipoprotein(a) in atherosclerotic cardiovascular disease and aortic stenosis: a European atherosclerosis society consensus statement. Eur Heart J. (2022) 43(39):3925–46. 10.1093/eurheartj/ehac361 36036785 PMC9639807

[20] Graham I Atar D Borch-Johnsen K Boysen G Burell G Cifkova R European guidelines on cardiovascular disease prevention in clinical practice: executive summary: fourth joint task force of the European society of cardiology and other societies on cardiovascular disease prevention in clinical practice (constituted by representatives of nine societies and by invited experts). Eur Heart J. (2007) 28(19):2375–414. 10.1093/eurheartj/ehm316 17726041

[21] Olmastroni E Gazzotti M Arca M Averna M Pirillo A Catapano AL Twelve variants polygenic score for low-density lipoprotein cholesterol distribution in a large cohort of patients with clinically diagnosed familial hypercholesterolemia with or without causative mutations. J Am Heart Assoc. (2022) 11(7):e023668. 10.1161/JAHA.121.023668 35322671 PMC9075429

[22] Authors/Task Force Members; ESC Committee for Practice Guidelines (CPG); ESC National Cardiac Societies. 2019 ESC/EAS guidelines for the management of dyslipidaemias: lipid modification to reduce cardiovascular risk. Atherosclerosis. (2019) 290:140–205. 10.1016/j.atherosclerosis.2019.08.014 31591002

[23] Pang J Poulter EB Bell DA Bates TR Jefferson VL Hillis GS Frequency of familial hypercholesterolemia in patients with early-onset coronary artery disease admitted to a coronary care unit. J Clin Lipidol. (2015) 9(5):703–8. 10.1016/j.jacl.2015.07.005 26350818

[24] Watts GF Sullivan DR Poplawski N van Bockxmeer F Hamilton-Craig I Clifton PM Familial hypercholesterolaemia: a model of care for Australasia. Atheroscler Suppl. (2011) 12(2):221–63. 10.1016/j.atherosclerosissup.2011.06.001 21917530

[25] Correction to: 2018 AHA/ACC/AACVPR/AAPA/ABC/ACPM/ADA/AGS/APhA/ASPC/NLA/PCNA guideline on the management of blood cholesterol: a report of the American College of Cardiology/American Heart Association Task Force on clinical practice guidelines. Circulation. (2023) 148(7):e5. 10.1161/CIR.0000000000001172. Erratum for: *Circulation*. (2019) 139(25):e1082-143. PMID: 37579012. 37579012

[26] Willeit P Ridker PM Nestel PJ Simes J Tonkin AM Pedersen TR Baseline and on-statin treatment lipoprotein(a) levels for prediction of cardiovascular events: individual patient-data meta-analysis of statin outcome trials. Lancet. (2018) 392(10155):1311–20. 10.1016/S0140-6736(18)31652-0 30293769

[27] Emerging Risk Factors Collaboration; Erqou S Kaptoge S Perry PL Di Angelantonio E Thompson A White IR Lipoprotein(a) concentration and the risk of coronary heart disease, stroke, and nonvascular mortality. JAMA. (2009) 302(4):412–23. 10.1001/jama.2009.1063 19622820 PMC3272390

[28] Langsted A Nordestgaard BG. Lipoprotein(a) as part of the diagnosis of clinical familial hypercholesterolemia. Curr Atheroscler Rep. (2022) 24(4):289–96. 10.1007/s11883-022-01002-0 35107760

[29] Ray KK Vallejo-Vaz AJ Ginsberg HN Davidson MH Louie MJ Bujas-Bobanovic M Lipoprotein(a) reductions from PCSK9 inhibition and major adverse cardiovascular events: pooled analysis of alirocumab phase 3 trials. Atherosclerosis. (2019) 288:194–202. 10.1016/j.atherosclerosis.2019.06.896 31253441

[30] Schatz U Fischer S Müller G Tselmin S Birkenfeld AL Julius U Cardiovascular risk factors in patients with premature cardiovascular events attending the university of dresden lipid clinic. Atheroscler Suppl. (2019) 40:94–9. 10.1016/j.atherosclerosissup.2019.08.044 31818455

[31] Afanasieva OI Tyurina AV Klesareva EA Arefieva TI Ezhov MV Pokrovsky SN. Lipoprotein(a), immune cells and cardiovascular outcomes in patients with premature coronary heart disease. J Pers Med. (2022) 12(2):269. Published 2022 Feb 12. 10.3390/jpm12020269 35207757 PMC8876319

[32] Paquette M Bernard S Thanassoulis G Baass A. LPA genotype is associated with premature cardiovascular disease in familial hypercholesterolemia. J Clin Lipidol. (2019) 13(4):627–633.e1. 10.1016/j.jacl.2019.04.006 31103339

[33] Nakamura E Sato Y Iwakiri T Yamashita A Moriguchi-Goto S Maekawa K Asymptomatic plaques of lower peripheral arteries and their association with cardiovascular disease: an autopsy study. J Atheroscler Thromb. (2017) 24(9):921–7. 10.5551/jat.39669 28367862 PMC5587518

[34] van Dam-Nolen DHK van Egmond NCM Dilba K Nies K van der Kolk AG Liem MI Sex differences in plaque composition and morphology among symptomatic patients with mild-to-moderate carotid artery stenosis. Stroke. (2022) 53(2):370–8. 10.1161/STROKEAHA.121.036564 34983237 PMC8785521

[35] Adler AI Stevens RJ Neil A Stratton IM Boulton AJ Holman RR. UKPDS 59: hyperglycemia and other potentially modifiable risk factors for peripheral vascular disease in type 2 diabetes. Diabetes Care. (2002) 25(5):894–9. 10.2337/diacare.25.5.894 11978687

[36] Lu JT Creager MA. The relationship of cigarette smoking to peripheral arterial disease. Rev Cardiovasc Med. (2004) 5(4):189–93. PMID: 15580157. 15580157

[37] Sanchez Muñoz-Torrero JF Rico-Martín S Álvarez LR Aguilar E Alcalá JN Monreal M. Lipoprotein (a) levels and outcomes in stable outpatients with symptomatic artery disease. Atherosclerosis. (2018) 276:10–4. 10.1016/j.atherosclerosis.2018.07.001 30006322

[38] Volpato S Vigna GB McDermott MM Cavalieri M Maraldi C Lauretani F Lipoprotein(a), inflammation, and peripheral arterial disease in a community-based sample of older men and women (the InCHIANTI study). Am J Cardiol. (2010) 105(12):1825–30. 10.1016/j.amjcard.2010.01.370 20538138 PMC2888047

[39] Gurdasani D Sjouke B Tsimikas S Hovingh GK Luben RN Wainwright NW Lipoprotein(a) and risk of coronary, cerebrovascular, and peripheral artery disease: the EPIC-norfolk prospective population study. Arterioscler Thromb Vasc Biol. (2012) 32(12):3058–65. 10.1161/ATVBAHA.112.255521 23065826 PMC4210842

[40] Price JF Lee AJ Rumley A Lowe GD Fowkes FG. Lipoprotein (a) and development of intermittent claudication and major cardiovascular events in men and women: the Edinburgh artery study. Atherosclerosis. (2001) 157(1):241–9. 10.1016/s0021-9150(00)00719-x 11427227

[41] Forbang NI Criqui MH Allison MA Ix JH Steffen BT Cushman M Sex and ethnic differences in the associations between lipoprotein(a) and peripheral arterial disease in the multi-ethnic study of atherosclerosis. J Vasc Surg. (2016) 63(2):453–8. 10.1016/j.jvs.2015.08.114 26518096

[42] Pradhan AD Shrivastava S Cook NR Rifai N Creager MA Ridker PM. Symptomatic peripheral arterial disease in women: nontraditional biomarkers of elevated risk. Circulation. (2008) 117(6):823–31. 10.1161/CIRCULATIONAHA.107.719369 18227386

[43] Ribatti D Vacca A Giacchetta F Cesaretti S Anichini M Roncali L Lipoprotein (a) induces angiogenesis on the chick embryo chorioallantoic membrane. Eur J Clin Invest. (1998) 28(7):533–7. 10.1046/j.1365-2362.1998.00322.x 9726032

[44] Xue J Liao Q Luo M Hua C Zhao J Yu G Cigarette smoke-induced oxidative stress activates NRF2 to mediate fibronectin disorganization in vascular formation. Open Biol. (2022) 12(4):210310. 10.1098/rsob.210310 35472288 PMC9042581

[45] Haitjema S van Haelst STW de Vries JPM Moll FL den Ruijter HM de Borst GJ Time-dependent differences in femoral artery plaque characteristics of peripheral arterial disease patients. Atherosclerosis. (2016) 255:66–72. 10.1016/j.atherosclerosis.2016.10.039 27821353

[46] Tsimikas S Gordts PLSM Nora C Yeang C Witztum JL. Statin therapy increases lipoprotein(a) levels. Eur Heart J. (2020) 41(24):2275–84. 10.1093/eurheartj/ehz310 31111151

[47] Yeang C Hung MY Byun YS Clopton P Yang X Witztum JL Effect of therapeutic interventions on oxidized phospholipids on apolipoprotein B100 and lipoprotein(a). J Clin Lipidol. (2016) 10(3):594–603. 10.1016/j.jacl.2016.01.005 27206947

[48] Yeang C Witztum JL Tsimikas S. ‘LDL-C'=LDL-C+Lp(a)-C: implications of achieved ultra-low LDL-C levels in the proprotein convertase subtilisin/kexin type 9 era of potent LDL-C lowering. Curr Opin Lipidol. (2015) 26(3):169–78. 10.1097/MOL.0000000000000171 25943842

[49] Jagannathan SN Connor WE Baker WH Bhattacharyya AK. The turnover of cholesterol in human atherosclerotic arteries. J Clin Invest. (1974) 54(2):366–77. 10.1172/JCI107772 4367889 PMC301564

[50] Kostakou PM Hatzigeorgiou G Kolovou V Mavrogeni S Kolovou GD. Lipoprotein (a) evolution: possible benefits and harm. Genetic and non-genetic factors influencing its plasma levels. Curr Med Chem. (2017) 24(10):969–78. 10.2174/0929867324666170120155412 28117004

[51] Mora S Kamstrup PR Rifai N Nordestgaard BG Buring JE Ridker PM. Lipoprotein(a) and risk of type 2 diabetes. Clin Chem. (2010) 56(8):1252–60. 10.1373/clinchem.2010.146779 20511445 PMC2912456

[52] Kostner KM Kostner GM. Lp(a) and the risk for cardiovascular disease: focus on the lp(a) paradox in diabetes Mellitus. Int J Mol Sci. (2022) 23(7):3584. 10.3390/ijms23073584 35408941 PMC8998850

[53] Liu T Yoon WS Lee SR. Recent updates of lipoprotein(a) and cardiovascular disease. Chonnam Med J. (2021) 57(1):36–43. 10.4068/cmj.2021.57.1.36 33537217 PMC7840349

[54] Liu HH Cao YX Jin JL Zhang HW Hua Q Li YF Predicting cardiovascular outcomes by baseline lipoprotein(a) concentrations: a large cohort and long-term follow-up study on real-world patients receiving percutaneous coronary intervention. J Am Heart Assoc. (2020) 9(3):e014581. 10.1161/JAHA.119.014581 32013705 PMC7033882

[55] Anderson TJ Grégoire J Pearson GJ Barry AR Couture P Dawes M 2016 Canadian cardiovascular society guidelines for the management of dyslipidemia for the prevention of cardiovascular disease in the adult. Can J Cardiol. (2016) 32(11):1263–82. 10.1016/j.cjca.2016.07.510 27712954

[56] Vinci P Di Girolamo FG Panizon E Tosoni LM Cerrato C Pellicori F Lipoprotein(a) as a risk factor for cardiovascular diseases: pathophysiology and treatment perspectives. Int J Environ Res Public Health. (2023) 20(18):6721. 10.3390/ijerph20186721 37754581 PMC10531345

[57] Molinari EA Pichler PF Grillhofer H Kostner GM. Immunoturbidimetric determination of lipoprotein(a): improvement in the measurement of turbid and triglyceride-rich samples. Clin Chim Acta. (1995) 235(1):59–69. 10.1016/0009-8981(95)06001-3 7634492

[58] Vinci P Panizon E Tosoni LM Cerrato C Pellicori F Mearelli F Statin-associated myopathy: emphasis on mechanisms and targeted therapy. Int J Mol Sci. (2021) 22(21):11687. 10.3390/ijms222111687 34769118 PMC8583847

[59] Biolo G Vinci P Mangogna A Landolfo M Schincariol P Fiotti N Mechanism of action and therapeutic use of bempedoic acid in atherosclerosis and metabolic syndrome. Front Cardiovasc Med. (2022) 9:1028355. 10.3389/fcvm.2022.1028355 36386319 PMC9650075

